# GFRAL Is Widely Distributed in the Brain and Peripheral Tissues of Mice

**DOI:** 10.3390/nu16050734

**Published:** 2024-03-04

**Authors:** Karoline Fichtner, Hermann Kalwa, Miao-Miao Lin, Yuanyuan Gong, Anne Müglitz, Michael Kluge, Ute Krügel

**Affiliations:** 1 Rudolf Boehm Institute of Pharmacology and Toxicology, Faculty of Medicine, University of Leipzig, D-04103 Leipzig, Germany; 2 School of Acupuncture and Tuina, Chengdu University of Traditional Chinese Medicine, Chengdu 610075, China; 3 Department of Psychiatry and Psychotherapy, University of Leipzig, D-04103 Leipzig, Germany; 4 Department of Psychiatry, Rudolf-Virchow-Klinikum Glauchau, D-08371 Glauchau, Germany

**Keywords:** GFRAL, brain, peripheral tissue, immunohistochemistry

## Abstract

In 2017, four independent publications described the glial cell-derived neurotrophic factor (GDNF) receptor alpha-like (GFRAL) as receptor for the growth differentiation factor 15 (GDF15, also MIC-1, NAG-1) with an expression exclusively in the mice brainstem area postrema (AP) and nucleus tractus solitarii (NTS) where it mediates effects of GDF15 on reduction of food intake and body weight. GDF15 is a cell stress cytokine with a widespread expression and pleiotropic effects, which both seem to be in contrast to the reported highly specialized localization of its receptor. This discrepancy prompts us to re-evaluate the expression pattern of GFRAL in the brain and peripheral tissues of mice. In this detailed immunohistochemical study, we provide evidence for a more widespread distribution of this receptor. Apart from the AP/NTS region, GFRAL-immunoreactivity was found in the prefrontal cortex, hippocampus, nucleus arcuatus and peripheral tissues including liver, small intestine, fat, kidney and muscle tissues. This widespread receptor expression, not taken into consideration so far, may explain the multiple effects of GDF-15 that are not yet assigned to GFRAL. Furthermore, our results could be relevant for the development of novel pharmacological therapies for physical and mental disorders related to body image and food intake, such as eating disorders, cachexia and obesity.

## 1. Introduction

Dysregulation in the control of food intake and body weight may lead to obesity or anorexia. Circulating high levels of the cell stress cytokine growth differentiation factor 15 (GDF15, also called MIC-1 and NAG-1) are linked to reduced appetite and food intake, loss of body weight and cancer-associated anorexia-cachexia [1,2,3,4]. In patients with obesity and type 2 diabetes mellitus, elevated GDF15 was found [5]. In mice models, GDF15 was effective in reducing food intake and adiposity and in counteracting metabolic dysfunction via increased energy expenditure [6,7]. GDF15 might exert its effects with multiple mechanisms, e.g., on food preference for non-fat diet, delay of gastric emptying, taste aversion or nausea and emesis [8].

In patients with anorexia nervosa, high serum GDF15 levels were found and were probably related to weight loss in this group, but can be reversed via realimentation [9,10,11]. Furthermore, the anti-diabetic drug metformin was shown to increase circulating GDF15 levels as a requirement for seeing beneficial effects regarding energy intake and expenditure, and weight loss, but not for lowering glucose [7].

The pleiotropic from of GDF15 is a distant member of the transforming growth factor β (TGF-β) family [12] that is widely expressed in, for example, the placenta, bladder, liver, kidney, epithelia of exocrine glands and brain [13,14,15]. Its synthesis is regulated by cell activation and cell stress involving p53, EGR-1 and transcription factors of the integrated stress response pathway [16]. Consequently, circulating GDF15 levels rise under ‘non-homeostatic’ conditions of adiposity, starvation, mitochondrial dysfunction, and insulin resistance, but also in inflammatory diseases and various cancer types [1,16].

In 2017, four research groups independently described that GDF15 binds with high affinity to the glial cell-derived neurotrophic factor (GDNF) family receptor alpha-like (GFRAL) and that this receptor has a strongly restricted expression in the brainstem [1,2,17,18].

GFRAL shares significant structural characteristics with the canonical members of the GDNF receptor family (GFR-α1-4), i.e., a highly conserved second cysteine-rich domain, a highly hydrophobic *N*-terminal signal peptide and its membrane anchorage [19]. However, in contrast to its distant homologs, a GPtdIns cleavage site at its *C*-terminal sequence is replaced by a single transmembrane region. Sequence analysis of GFRAL indicated that only 30% of its amino acid sequence is identical to that of GFRα-3 [19]. The distant relation of GFRAL from the GFR-α family is also apparent in specific ligand binding. Whereas recombinant native GDNF binds to the GFR-α1 and 2 isoforms, it does not interact with GFRAL. Otherwise, GDF15 exclusively binds to GFRAL but not to its homologs GAS-1 or GFR-α1-3 [18].

Activation of GFRAL via GDF15 recruits the tyrosine kinase rearranged during transfection (RET) as a co-receptor stimulating intracellular downstream pathways, e.g., extracellular signal-regulated kinase (ERK), protein kinase B (AKT) and phospholipase Cγ (PLCγ) [16]. The unique action of GDF15 on GFRAL at area postrema/nucleus tractus solitarii (AP/NTS) neurons is suggested to activate neurons in the parabrachial nucleus (PBN)-central amygdala circuit to mediate food and taste aversion, GDF15 driven anorexia and weight loss [1,2,20,21]. Thereby, the GDF15/GFRAL/RET signaling pathway represents a potential therapeutic target for eating disorders, metabolic diseases and cancer-related cachexia [2,22,23,24].

However, to date, the biological function of the GDF15/GFRAL system is poorly understood, i.e., whether elevated GFRAL signaling directed to restore homeostasis might be ineffective or even harmful in severe disease [16,25].

In fact, a further uncertainty is the widespread expression of GDF15 and its apparently pleiotropic actions [26], particularly in ‘non-homoestatic’ disease conditions, while its receptor GFRAL is described to be strongly restricted to the AP/NTS. An obvious explanation for this might be that there is a more extensive receptor distribution than is currently presumed at low levels, but biologically sufficient levels that evaded consideration so far.

A review of the previous literature provided some hints for such a more widespread GFRAL expression. For example, Li et al. (2005) found strong GFRAL transcripts, e.g., in the substantia nigra and the hippocampus of adult mice [19]. Apart from a high expression in the brainstem, Mullican et al. (2017) also showed low GFRAL mRNA expression in the hippocampus and cortex of mice. Further, they described strong GFRAL mRNA expression in the human testes and adipose tissues and various other human peripheral tissues also expressed GFRAL, albeit at low levels [2]. Finally, gene expression data from the GTEx portal suggest that GFRAL protein expression could be more ubiquitous distributed, although in small quantities [27].

These discrepancies to the commonly cited highly specialized receptor localization prompted us to re-evaluate the expression pattern of GFRAL in healthy mice by utilizing immunofluorescence labeling of GFRAL. For this purpose, we referred to the polyclonal sheep anti-GFRAL antigen affinity-purified antibody used by the first researchers that described its use, as well as others [1,22].

With this detailed analysis on the cellular level, in addition to the commonly accepted GFRAL expression in the AP/NTS, GFRAL-immunoreactivity (IR) was found in further brain areas and peripheral tissues. This observation is relevant for the understanding of disorders related to food intake, body image and their therapies.

## 2. Materials and Methods

### 2.1. Animals

Animal experiments were performed according to the ARRIVE guidelines, to the European (Council Directive 2010/63/EU) and to the German guidelines for the welfare of experimental animals. Experiments were approved by the local authorities (Landesdirektion, Leipzig, Germany). Male and female wild-type C57BL/6J mice (10–12 weeks old, 23–25 g, Charles River, Sulzfeld, Germany) were housed under an air-conditioned standard environment with free access to food and water and a 12 h light/dark cycle (lights on from 7:00 a.m.). A total of 4 male and 4 female mice were randomly selected; to the best of our knowledge, no gender differences in naïve animals were described. Mice were euthanized with an overdose of isoflurane and transcardially rinsed with 10 mL of heparinized saline, followed by 10% neutral buffered formalin.

### 2.2. Immunofluorescence Labeling and Confocal Laser Scanning Microscopy

The brain, the medial lobe of liver, visceral perigonadal white fat tissue, duodenum tissue, medulla of kidney, heart and skeletal muscle and the aorta were dissected, post-fixed in 4% paraformaldehyde (PFA) overnight, dehydrated and embedded in paraffin. Tissue sections of 6 µm thickness were cleared from paraffin and treated with citrate buffer pH 6 at 85 °C for 15 min to unmask antigenic epitopes.

Unspecific binding was blocked with 5% bovine serum albumin and 0.3% Triton in PBS for 1 h. Slices were incubated over night at 4 °C with the following primary antibodies: sheep anti-GFRAL (1:200, R&D Systems #AF5728, Lot. CDBG062305A, Minneapolis, MN, USA) and, additionally for brain, mouse anti-MAP2 (microtubule associated protein 2, a neuron specific cytoskeletal protein) (1:1000, Abcam #ab254144, Lot. GR3390046-5, Cambridge, UK).

Sections were washed with PBS followed by incubation for 2 h at room temperature with the respective secondary antibodies. For GFRAL, donkey anti-sheep IgG (H+L) cross-adsorbed secondary antibodies (1:1000, Alexa Fluor™ 680, ThermoFisher Scientific #A-21102, Waltham, MA, USA) were used. MAP2 antibodies were detected with donkey anti-mouse IgG (H+L) highly cross-adsorbed secondary antibody (1:1000, Alexa Fluor™ 488, ThermoFisher Scientific #A-21202). In negative controls, the GFRAL antibody was omitted. Sections were washed again.

In a control experiment for hippocampal expression, slices were incubated alternatively with rabbit anti-GFRAL (1:500, antibodies-online #ABIN2174394, Lot. A106214490, Limerick, PA, USA). For these slices, blocking was performed with 3% milk powder, 5% goat serum and goat anti-rabbit IgG (H+L) unconjugated (1:200, VectorLabs #AI-1000, Newark, CA, USA) in TBS was used. To counterstain GFRAL-IR against glycoproteins of the plasma membrane, wheat germ agglutinin (WGA) Alexa Fluor 488 conjugated (1:200, ThermoFisher Scientific W11261) was incubated for 2 h at room temperature followed by washing with TBS and incubation with goat anti-rabbit IgG (H+L) cross-adsorbed secondary antibody, Alexa Fluor™ 633 #A-21070. Sections were washed again.

Finally, all slices were rinsed with distilled water before the addition of Fluoromount-G™ Mounting Medium, containing DAPI to label DNA in the nuclei (ThermoFisher Scientific #00-4959-52). For imaging, a confocal live cell microscope Leica TCS SP8/DMi8 with the LAS X Software (version 3.5.7.23225, Leica Biosystems, Wetzlar, Germany) was used. Each experiment was performed in independent triplicates. Figures were arranged with the graphic software CorelDRAW 2022.

### 2.3. Antibody Validation with HFF-1 and HEK293 Cells

In addition to the reported proofs, the specificity of the sheep anti-GFRAL antibody was verified using two cell lines, human foreskin fibroblasts (HFF-1) and human embryonic kidney (HEK293) cells—negative and positive for GRFAL mRNA, respectively (for methods and results, see the Supplementary Materials section and Figure S1).

## 3. Results

### 3.1. GFRAL-IR Is Expressed in Various Mouse Brain Areas

In the brains of mice, immunofluorescence data confirmed the former descriptions on GFRAL in the brainstem. In addition, it was detectable in other brain areas.

**Figure 1:**
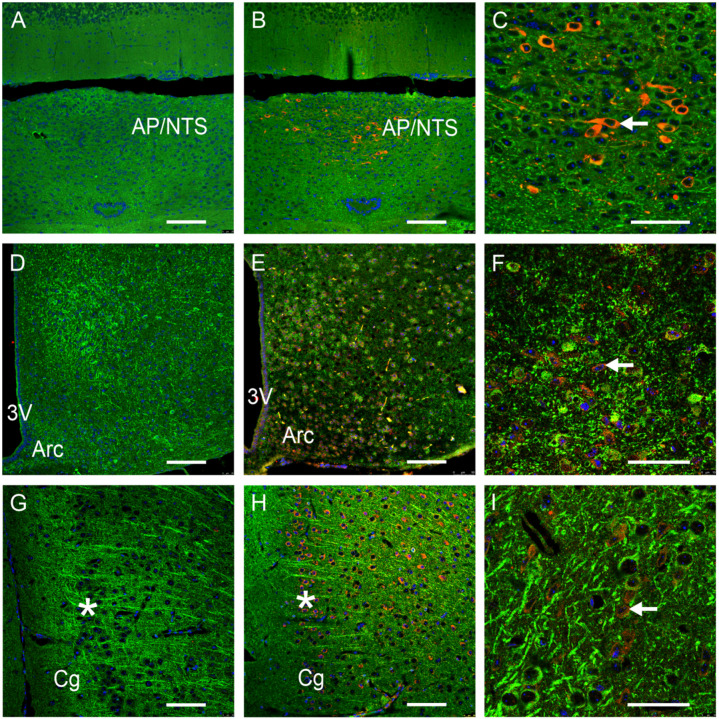

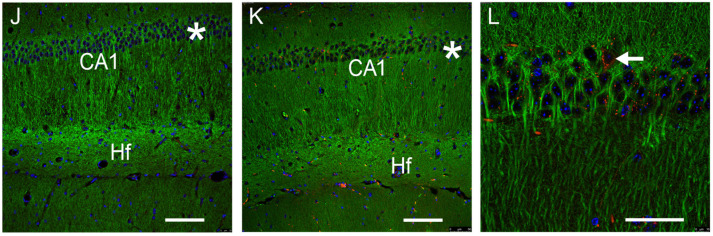
GFRAL-immunoreactivity (IR) in various brain areas of mice. Colors represent GFRAL-IR structures (red), nuclei (blue) and microtubule-associated protein 2 (MAP2, green). Negative controls are shown in (**A**,**D**,**G**,**J**). Area postrema (AP) and nucleus tractus solitarii (NTS) (**A**–**C**); nucleus arcuatus (Arc, (**D**–**F**)); prefrontal cortex (**G**–**I**); dorsal hippocampus (CA1 region, (**J**–**L**)); 3V: third ventricle; Cg: cingulate cortex; Hf: hippocampal fissure. *: pyramidal cell layer, ←: GFRAL-IR-positive cell. Scale bars: 100 μm in (**A**,**B**,**D**,**E**,**G**,**H**,**J**,**K**) (20×) and 50 μm in (**C**,**F**,**I**,**L**) (63×).

In detail, strong GFRAL-IR-positive cells were found as expected in the AP/NTS region (Figure 1). A much lower GFRAL antibody specific for IR with less dense distribution could be detected in the nucleus arcuatus, a part of the hypothalamus (Figure 1). In slices from the medial prefrontal cortex, GFRAL-positive cells were observed in particular in layer 2/3 of the cingulate and prelimbic part, but also diffusely distributed in the deeper cortex (Figure 1). Very weak GFRAL-IR further appeared in the pyramidal cell layer of the CA1 region in the dorsal hippocampus (Figure 1), which could hardly be specifically assigned to a cellular structure.

**Figure 2:**
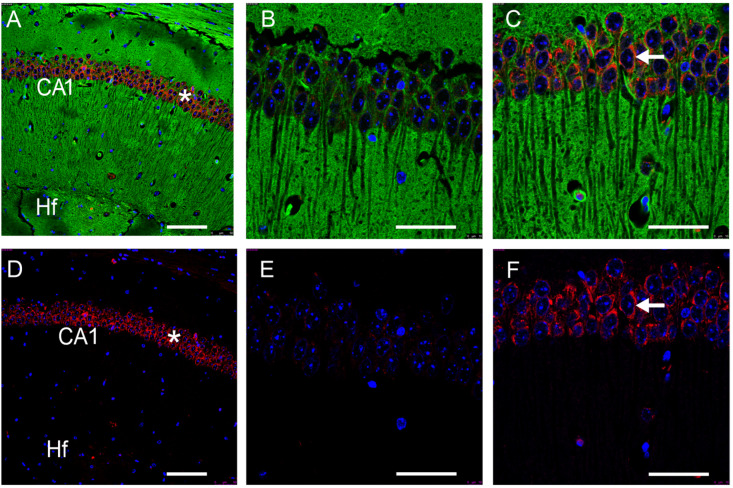
Verification of GFRAL-immunoreactivity (IR) in the hippocampus of mice. Colors represent GFRAL-IR structures (red), nuclei (blue) and wheat germ agglutinin (WGA, green). Negative controls are shown in (**B**,**E**). CA1: hippocampal CA1 region; Hf: hippocampal fissure; *: pyramidal cell layer; ←: GFRAL-IR-positive cell. Scale bars: 100 μm (20×) in (**A**,**D**) and 50 μm (63×) in (**B**,**C**,**E**,**F**).

Therefore, a control experiment using a rabbit anti-GFRAL antibody labeled against WGA was performed. In this approach, GFRAL-IR in the hippocampal pyramidal cell layer could conclusively be proven with an appearance compatible with a membrane-bound protein (Figure 2).

### 3.2. Peripheral Tissues of Mice Express GFRAL-IR

**Figure 3:**
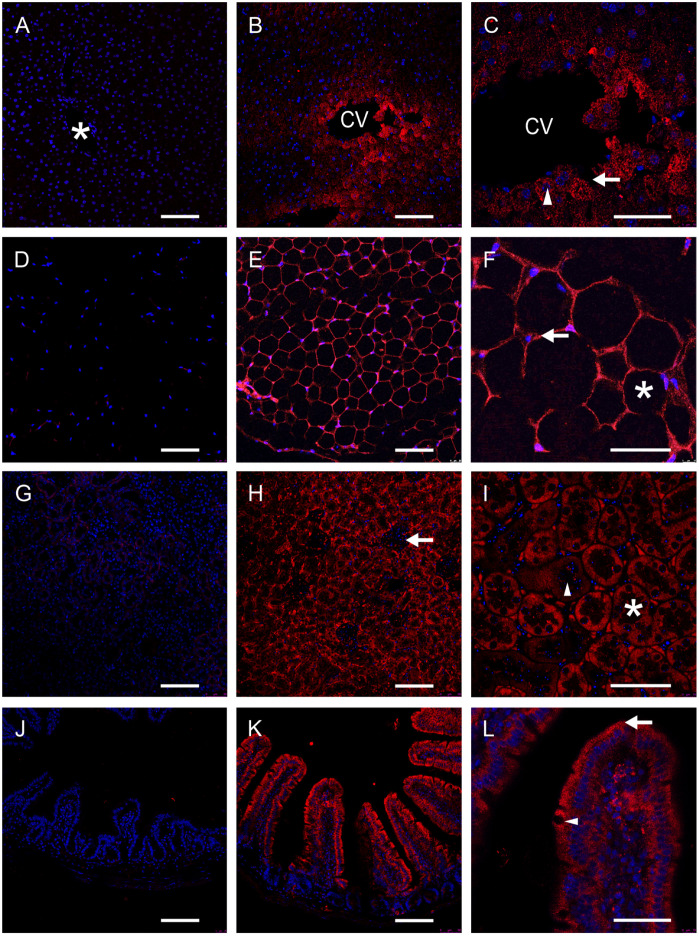
GFRAL-immunoreactivity (IR) in peripheral tissues of mice. In the liver (**A**–**C**), fat tissue (**D**–**F**), kidney (**G**–**I**) and intestine (**J**–**L**); GFRAL-positive structures appear in red and nuclei in blue. Negative controls are shown in (**A**,**D**,**G**,**J**). Liver (**A**–**C**): CV: central vein; *: positive hepatocytes; ▲: hepatocyte; ←: sinusoid. Visceral fat (**D**–**F**): *: fat reservoir; ←: cytoplasm of adipocytes. Kidney (**G**–**I**): ←: renal corpuscle; *: proximal tubulus; ▲: distal tubulus. Small intestine (**J**–**L**): ←: mucosa cells with luminal brush border; ▲: goblet cell. Scale bars: 100 μm in (**A**,**B**,**D**,**E**,**G**,**H**,**J**,**K**) (20×) and 50 μm in (**C**,**F**,**I**,**L**) (63×).

GFRAL-IR was found to be widely distributed in peripheral tissues. GFRAL-positive hepatocytes were particularly observed around the central vein (Figure 3A–C). In the visceral fat tissue, GFRAL-labeling appears densely around the fat reservoir along the membrane (Figure 3D–F). GFRAL-IR was found all over the kidney, probably in the proximal tubulus cells in the parts associated with their brush border. The interior of distal tubuli and the renal corpuscles were free of GFRAL-IR (Figure 3G–I). In the intestine, cells of the mucosa (enterocytes) were also fluorescence-labelled for GFRAL, particularly the microvilli. Possibly, the IR-negative gaps at the luminal mucosal surface are attributable to goblet cells (Figure 3L).

**Figure 4:**
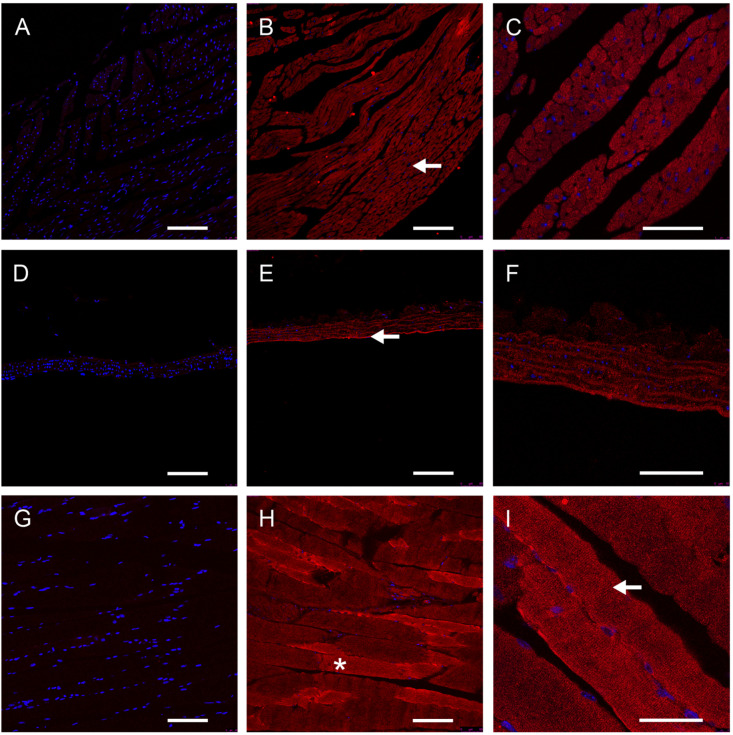
Immunohistochemical presentation of GFRAL-positive structures in the muscles of mice. GFRAL-positive structures (red); nuclei (blue). Negative controls are shown in (**A**,**D**,**G**). Heart muscle (**A**–**C**): ←:striation. cardiac muscle fibers. Aorta (**D**–**F**): ←: tunica. Skeletal muscle (**G**–**I**): * skeletal muscle fibers; ←: striation. Scale bars: 100 μm (20×) in (**A**,**B**,**D**,**E**,**G**,**H**), and 50 μm (63×) in (**C**,**F**,**I**).

GFRAL-positive elements were detected in the striated heart and skeletal muscle but also in the smooth muscle of the vascular wall of the aorta (Figure 4).

## 4. Discussion

In summary, we showed that GFRAL is much more widely expressed in mice than is currently considered. In agreement with previous reports, high GFRAL-IR expression was found in the neurons of the AP/NTS, which is described to modulate the anorectic effects of GDF15 [1,2,17,18]. Further, this study proved that GFRAL-IR is present in the cells of the nucleus arcuatus and the pyramidal cell layers of the prefrontal cortex and hippocampus. Moreover, in selected peripheral tissues, GFRAL-IR was apparent in the liver, visceral fat tissues, kidneys, mucosa of the intestines and muscular structures.

The technique used based on the same polyclonal sheep anti-GFRAL antigen affini-ty-purified antibody originally used by the first researchers that used it, as well as others [1,22]. This antibody did not bind to GFRAL-related proteins in ELISA and failed to label GFRAL in the brainstem of GFRAL-knockout mice [1]. Here, the antibody was further validated in two cell lines proved to be negative or positive for GFRAL transcripts. HEK293 cells were specifically labeled, and no unspecific IR was detectable (see the Supplementary Materials). Li et al. (2005) described an isoform of GFRAL missing the transmembrane region that was suggested to represent a soluble form of GFRAL with a still-unknown function, which may be recognized by the used antibody as well [19]. Of note, the tissue studied here was permeabilized, a process which may interfere with an expected strong membrane-associated IR [19].

This study confirmed the intensive labeling of AP/NTS neurons, whereas the GFRAL-IR in other brain regions appeared to be specific, but weaker, and therefore may have evaded their detection so far.

For the AP/NTS region, the expression of GFRAL is conserved in rodents, monkeys and human [18]. In the other analyzed brain structures, the cellular GFRAL-IR is consistent with the GFRAL transcript distribution in adult mice, which is particularly reported for the cortex and hippocampus, although it is comparatively low [2,19]. Both groups could not detect GFRAL in peripheral tissues using RT-PCR analysis in adult mice, which might be attributable to a rare expression not recognizable via bulk PCR analysis [23]. However, in a number of human brain regions and peripheral tissues, GFRAL transcripts were detectable on a low scale, with the exception of high levels found in fat tissues, testes and mammary tissues [2,27].

The distribution of GFRAL-IR found here matches the widespread expression of its ligand, GDF15, in the brain and peripheral tissues of mice and humans [13,15,28].

Consequently, this challenges the notion that the circulating pleiotropic GDF15 exclusively activates GFRAL-expressing neurons in the AP/NTS, thereby being involved in anorexia and weight loss [4,16,23].

Rather, our immunohistochemical data point out the possibility that GFRAL is also activated in other brain areas and peripheral tissues via local or circulating GDF15. Here, GDF15/GFRAL signaling could be involved in keeping or restoring cellular homeostasis in disease or aging, as outlined below.

Similar to the AP/NTS, as a circumventricular organ with vascular permeability, neurons of the hypothalamic arcuate nucleus—a major center for appetite control—are accessible for GDF15. These neurons are open to the cerebrospinal fluid in the infundibular recess of the third ventricle [29,30]. Notably, GDF15 is also synthesized in the choroid plexus and secreted into the ventricle [14].

In the healthy mice in our study, GFRAL-IR in the arcuatus region was less intense compared to the AP/NTS, as were the GFRAL transcripts elsewhere [2]. This low expression may explain that their detection has failed using in situ hybridization analysis [18,31].

As present research mainly focuses on the exclusive role of GFRAL in the AP/NTS in anorexia, tumor-induced cachexia and obesity, the data on potential GDF15/GFRAL signaling in other brain areas are sparse. It has been consistently shown that GDF15 is synthesized in the cortex and hippocampus of mice and humans, and that it is highly regulated in the brain, for example, by ischemia, aging or Alzheimer’s disease [13,32,33].

The release of GDF15 occurs downstream to cell stress related to mitochondrial and endoplasmic reticulum dysfunction and is suggested to exert anti-inflammatory effects by enhancing tissue tolerance to inflammation, as well as through the counter-regulation of inflammation-induced expression of pro-inflammatory cytokines [12,33,34,35,36]. Although the physiological functions of GDF15 are not yet fully understood, its upregulation can be considered as an attempt to compensate for cell stress [33]. However, the receptors and signaling pathways by which brain-derived GDF15 may induce such local responses are still unclear [33].

The GFRAL-IR, in comparison to its reported low mRNA outside the AP/NTS of healthy adult mice, might suggest that GFRAL-mediated signaling is repressed in normo-physiological situations, e.g., for the control of appetite [24]. It should be noted that mRNA levels are not the final output of gene expression, but that they mechanistically represent the templates required for biological systems’ potential highly controlled protein synthesis on demand [37]. This implies the possibility of an upregulation of GFRAL under challenging conditions, like specific developmental stages in mice and humans [19,38] or obesity. The low or even missing detection of GFRAL transcripts apart from AP/NTS may have prevented the investigation of such demanding circumstances in more detail so far.

GFRAL transcripts, even very slight, are also noticed in the peripheral tissues of humans or occasionally in mice in the current databases, but are barely further reported [27,39]. Similar to what we have found in various brain areas, GFRAL-IR was also shown to be present in the peripheral parts of the liver, visceral fat tissues, kidneys, mucosa of the intestines and muscular structures.

Our results in mice were confirmed with the detection of GFRAL-IR in human normal gastric mucosa as well as in gastric cancer cells [40]. Interestingly, in the latter GDF15, GFRAL and RET proteins are significantly and jointly elevated. Similarly, GFRAL-IR was detected in human normal pancreatic ductal epithelial and cancer cells, which was positively correlated with GDF15 expression [41]. Furthermore, in the dual immunofluorescence analysis of pancreatic cancer cells, both proteins were co-expressed. Although it was not shown whether this co-expression is also existent in normal tissues, this constellation strongly argues for carrying out feedback sensing of autocrine-released GDF15 from pancreatic cancer cells. On the other hand, pancreas carcinoma and pancreatitis are strongly associated with nausea, reduced food intake and anorexia/cachexia, and are obviously mediated by the GDF15/GFRAL brainstem pathway. The autocrine or endocrine properties of GDF15 have been discussed repeatedly, particularly regarding the adjustment of metabolic activity, and may be in line with the reported enhancement of GDF15 expression in the liver, heart and muscles after physical exercise, as reviewed by Wang (2021) [4,8,20,42,43]. However, at present, the identity of a respective receptor remains unclear, but the current data suggest that GFRAL might be a candidate.

In view of the shown GFRAL distribution in mice—results obtained with full GFRAL-KO mice or with the use of blocking antibodies should be interpreted with care. Nevertheless, full or cell-specific KO mice as well as the use of adeno-associated viruses (AAV) to assess gene function in or beyond AP/NTS are important research tools to further explore the role of GFRAL in the brain and peripheral organs under disease conditions. Although no functional investigations were performed, a more global regulatory role of GFRAL in disorders related to food intake, such as eating disorders, anorexia/cachexia and obesity, is conceivable.

In genome-wide association studies (GWAS) on variants, a GFRAL missense variant, rs12199003 (p.Arg33Cys), which obviously causes impaired binding to GDF15, was associated with BMI [8,17,44]. Further, in a transcriptome wide analysis (TWAS), GFRAL expression in human adipose tissue was negatively associated with higher levels of the orexigenic peptide ghrelin, mediating several further opposing effects to the GDF15/GFRAL signaling [45]. Interestingly, a gene variant of GFRAL was found using next-generation sequencing in a cohort of patients exhibiting diverse clinical signs of anorexia nervosa; however, its clinical relevance is unknown [46].

## 5. Conclusions

More insights into the genetic variants of GFRAL—together with a more comprehensive knowledge about the control of the transcription and translation of GFRAL under disease conditions, as well as further explanations of GDF15-mediated effects beyond the AP/NTS—could be relevant for the development of novel pharmacological therapies for physical and mental disorders related to body image and food intake, such as eating disorders, cachexia and obesity.

## Data Availability

The original contributions presented in this study are available upon request from the corresponding author.

## Supplementary Materials

The following supporting information can be downloaded at: https://www.mdpi.com/article/10.3390/nu16050734/s1, Figure S1: Antibody validation for GFRAL in HEK and HFF1 cells.
