# Supplementary material for: GFRAL Is Widely Distributed in the Brain and Peripheral Tissues of Mice
Source: Nutrients. 2024 Mar 4;16(5):734. doi: 10.3390/nu16050734 (PMC10933958; doi:10.3390/nu16050734)
Supplement: Supplementary file 1 [file nutrients-16-00734-s001.zip › nutrients-2869124-supplementary.pdf]

nutrients

Supplementary material

GFRAL Is Widely Distributed in the Brain and Peripheral Tissues of Mice

Karoline Fichtner [1,†], Hermann Kalwa [1,†], Miao-Miao Lin [1], Yuanyuan Gong [1,2] , Anne Müglitz [1] , Michael Kluge [3,4,‡] and Ute Krügel [1,*,‡]

[1] Rudolf Boehm Institute of Pharmacology and Toxicology, Faculty of Medicine, University of Leipzig, D-04103 Leipzig, Germany
[2] School of Acupuncture and Tuina, Chengdu University of Traditional Chinese Medicine, Chengdu 610075, China
[3] Department of Psychiatry and Psychotherapy, University of Leipzig, D-04103 Leipzig, Germany
[4] Department of Psychiatry, Rudolf-Virchow-Klinikum Glauchau, D-08371 Glauchau, Germany
* Correspondence: ute.kruegel@medizin.uni-leipzig.de
† These authors contributed equally to this work.
‡ These authors contributed equally to this work.

Antibody validation with HFF-1 and HEK293 cells

1. Method

The human embryonic kidney (HEK) 293 cell line was cultured from frozen stocks at 37 °C and 5% CO2 in Dulbecco's Modified Eagle Medium (DMEM; Capricorn Scientific GmbH, Germany) supplemented with 10% (v/v) fetal calf serum (Biochrom, Germany), 2 mM L-glutamine (Invitrogen GmbH, Deutschland), 100 U ml−1 penicillin G, 110 mg l−1 sodium pyruvate and 100 µg ml−1 streptomycin (Sigma, Germany).

The human foreskin fibroblasts (HFF-1)-1cell line was cultured from frozen stocks at 37 °C in RPMI 1640 Medium (Roswell Park Memorial Institute 1640 Medium (Sigma, Germany) supplemented with 10% heat-inactivated fetal bovine serum (FBS Superior, Biochrom, Germany). All lines were routinely split twice a week, when ~80% confluency was reached.

The RNA was extracted using TRI Reagent® (Sigma, Germany) according to the manufacturer's instructions. The obtained RNA pellet was dissolved in 30 µl DEPC H2O. Using NanoDrop™ 2000, the RNA concentration was determined in 2 µl solution each. The corresponding cDNA was synthesized from 1 µg of RNA samples using the Thermoscientific RevertAid H Minus First Strand cDNA Synthesis Kit (ThermoFisher Scientific, USA). The thermal cycler protocol was: 65 °C for 5 min, 25 °C for 5 min, 42 °C for 1 h and 70 °C for 5 min. The RT-qPCR products were pipetted onto 96-well plates using the Thermoscientific DyNAmo ColorFlash SYBR® Green qPCR Kit (ThermoFisher Scientific, USA), per batch: 4 µl cDNA, 1 µl sense and antisense primer pair, 5 µl SYBR® Green master mix. Primers used were for GFRAL: forward 5′-TTCCTGGCTGTTACGTTAAGC-3′, reverse 5′-GCCATTTGCATCAATCAAGCA-3′ and for the housekeeper PGK1: forward primer 5′-GCAGATTGTTTGGAATGGTC-3′, reverse primer 5′-TGCTCACATGGCTGACTTTA-3′. The reaction was carried out using the Applied Biosystems StepOnePlus Real-Time PCR System. The melting curves were recorded to exclude sequence deviations or mutations. To test the amplified PCR products for their purity and correct length, agarose gel electrophoresis was performed.

For immunofluorescence validation the cells were transferred to a cell culture dish

with medium for one day. The medium was removed and the cells were completely covered with 4% PFA for 15 minutes, rinsed with PBS and treated with the blocking solution for the regular subsequent immunofluorescence staining protocol with the sheep anti-GFRAL (1:200, R&D Systems #AF5728, Lot. CDBG062305A) as described in the main part.

2. Results

The validation of the sheep anti-GFRAL antibody used to examine the receptor distribution in mice revealed that HFF-1 cells shown not to express GFRAL mRNA (Fig. S1A, B) are also negative for GFRAL-IR. HEK293 cells, which express mRNA, appeared immunoreactive for GFRAL.

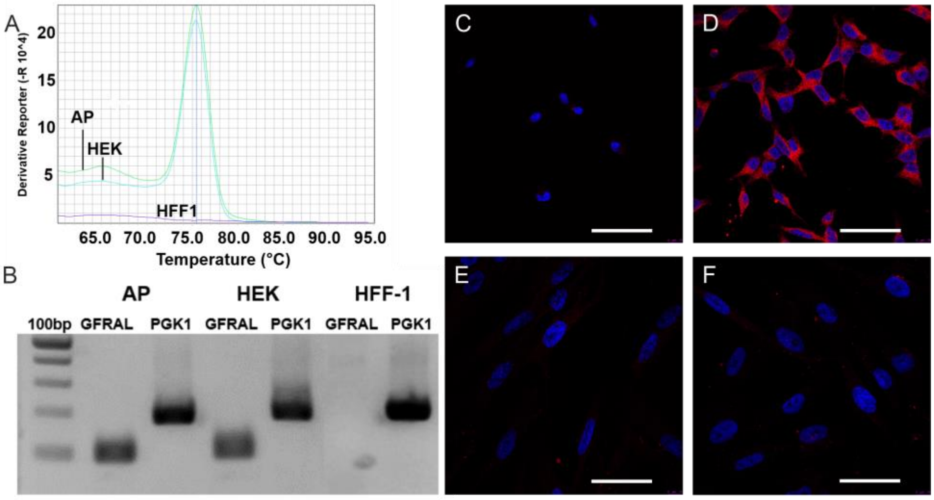

Figure S1 Antibody validation for GFRAL in HEK293 and HFF-1 cells. A melting curve analysis (from 60 °C to 95°C) (A) and a subsequent agarose gel electrophoresis (B) revealed no products for GFRAL in preparations from HFF-1 cells, but in HEK293 suspensions and in mouse reference tissue from the area postrema/ nucleus tractus solitarii (AP). As housekeeper phosphoglycerate kinase (PGK1) was used. In immunofluorescence images from HEK293 (C, D) and HFF-1 cells (E, F) GFRAL-immunoreactivity (red) and DAPI for nuclei (blue) are shown. Negative controls: C and E. No unspecific immunoreactivity is present in HFF-1 cells. Scale bars: 50 µm (20x)
